# Diagnostic Workup in IgE-Mediated Allergy to Asteraceae Weed Pollen and Herbal Medicine Products in Europe

**DOI:** 10.3390/medicina60091494

**Published:** 2024-09-13

**Authors:** Mariana Preda, Sylwia Smolinska, Florin-Dan Popescu

**Affiliations:** 1 Faculty of Medicine, Department of Allergology “Nicolae Malaxa” Clinical Hospital, “Carol Davila” University of Medicine and Pharmacy, 022441 Bucharest, Romania; mariana.preda@umfcd.ro; 2 Faculty of Medicine, Department of Clinical Immunology, Wroclaw Medical University, 51-616 Wroclaw, Poland; sylwia.smolinska@umw.edu.pl

**Keywords:** allergy diagnosis, Asteraceae pollen, herbal products

## Abstract

Anemophilous weeds from the Asteraceae family are highly allergenic and represent a significant source of aeroallergens in late summer and autumn. Ragweed and mugwort pollen allergies have become a significant health burden in Europe. Some people with respiratory allergies to weed pollen may also suffer hypersensitivity reactions to herbal medicines obtained from certain cross-reactive plants in the Compositae family, such as chamomile, marigold, and purple coneflower. General physicians, ear, nose, and throat (ENT) specialists, and pulmonologists need to be familiar with the diagnostic tests used by allergists in clinical practice to support accurate diagnosis in such patients. Allergists must also be aware of the suggestions of the European Medicines Agency (EMA)’s Herbal Medicinal Products Committee and the broad spectrum of herbal therapies to educate their patients about potential risks.

## 1. Introduction

The Asteraceae family, also known as Compositae, consists of over 25,000 species worldwide and is known for its wide variety of flowering plants in Europe. Some highly allergenic species, such as ragweed and mugwort, are anemophilous (wind-pollinated) weeds that produce large amounts of airborne pollen, which can be significant sources of aeroallergens in late summer and fall, especially in regions where these weeds are abundant but also outside the local pollination areas due to atmospheric long-distance transport. Currently, more than 15 million people in Europe have clinically significant sensitization to ragweed pollen alone, and the worst projections suggest that sensitization to this weed pollen will more than double by 2060 [1,2,3,4]. However, most Asteraceae species are entomophilous, and their composite flower heads are particularly effective at facilitating insect pollination. Some patients with weed pollen respiratory allergy may face risks of IgE-mediated hypersensitivity adverse reactions when consuming apitherapeutic products, such as artisanal honey and bee pollen as a food supplement, because such edible products may contain many pollen grains from some Asteraceae plants, like dandelion and sunflower, which can cross-react with those from allergenic anemophilous weeds. Similar risks exist with herbal products derived from the aerial parts and flowers of Compositae plants, such as camomile, marigold, and purple coneflower, used for their phytotherapeutic compounds [5,6,7,8].

## 2. IgE-Mediated Allergy to Asteraceae Weed Pollen

The most important Asteraceae pollen grains responsible for late summer-autumn seasonal respiratory allergies in Europe are produced by common/short ragweed (*Ambrosia artemisiifolia*) and common mugwort (*Artemisia vulgaris*), weeds with ruderal and segetal distribution. In the nineteenth century, common ragweed was accidentally introduced in Europe as a seed contaminant. *Ambrosia artemisiifolia* (NCBI: txid4212) has a heterotypic synonym *Ambrosia paniculata*, while *Ambrosia artemisiifolia* var. *elatior* (NCBI: txid4215) has the homotypic synonym *Ambrosia elatior*. Other invasive *Ambrosia* species in Europe are western ragweed (*Ambrosia psilostachya*) and giant ragweed (*Ambrosia trifida*). Besides the largely distributed common mugwort, other species grow in Europe and are represented by white mugwort (*Artemisia alba*), Chinese mugwort (*Artemisia verlotiorum*), common wormwood (*Artemisia absinthium*), and sweet wormwood (*Artemisia annua*), all with recognized ethnobotanical tradition. Sunflower (*Helianthus annuus*) is primarily grown for commercial use, while uncultivated wild plants may be considered weeds. *Artemisia* spp. belong to the Anthemideae tribe in the Asteroideae subfamily, part of the Asteraceae family, while *Helianthus* and *Ambrosia* spp. belong to the Heliantheae tribe of the same subfamily and family [1,2,9,10].

The clinical spectrum of IgE-mediated allergies to Asteraceae weed pollen varies from seasonal rhinitis/rhinoconjunctivitis to severe asthma. Because ragweed and mugwort pollen have a high degree of allergenic cross-reactivity, and in many parts of Europe, the distribution and pollination of mugwort and ragweed overlap, an accurate allergy diagnosis may be challenging [1,11].

Ragweed and mugwort pollen aeroallergens have molecular components represented by defensin-like proteins linked to polyproline-rich region (defensin-polyproline-linked proteins) Art v 1 and Amb a 4, pectate lyases Amb a 1 and Art v 6, cysteine protease Amb a 11, enolase Amb a 12, superoxide dismutase Amb t 13, triosephosphate isomerase Amb t 18, type 1 non-specific lipid transfer proteins (nsLTPs) Art v 3 and Amb a 6, pathogenesis-related protein PR-1 Art v 2, plastocyanines Amb a 3 and Amb a 7, group 5 allergens Amb a 5, Amb p 5 and Amb t 5, polcalcins Art v 5, Amb a 9 and Amb a 10, profilins Art v 4, Amb a 8 and Amb t 8 [12]. It should be emphasized that some ragweed pollen molecules have protease activity that can degrade tight junction proteins in the airway epithelial cells, thus facilitating allergenicity [13]. In addition, mugwort pollen is a significant vector for airborne endotoxin lipopolysaccharides, essential factors for inflammatory responses and allergic sensitization. Such endotoxins also adhere to ragweed pollen [14].

It is also relevant to highlight that cross-reactivity between Asteraceae aeroallergens and some food allergens in patients with weed pollen allergy may be associated with IgE-mediated hypersensitivity symptoms ranging from oral allergy syndrome to severe anaphylaxis. Many pollen-food syndromes and associations were described, such as celery-mugwort-spice syndrome and mugwort-peach, mugwort-mustard, and ragweed-melon-banana associations. Cross-reactive allergen components may be defensin-like proteins, nsLTPs, profilins, and high-molecular-weight allergens [6,15]. Interestingly, there are only several non-food exposure sources of nsLTP allergens, such as *Platanus acerifolia*, *Cannabis sativa*, *Ambrosia artemisiifolia,* and *Artemisia vulgaris*, and only some pollen grains, such as those from mugwort and plane tree, are potentially playing a major role in sensitization to food nsLTPs [16].

## 3. IgE-Mediated Allergy to Asteraceae Herbal Medicine Products (HMPs)

Herbal medicine, with thousands of years of tradition, is still increasingly popular in Europe as a complementary and alternative medicine. The European Directive 2004/24/EC provides the basis for using HMPs in the EU. Their safety profile should be evaluated based on existing data from clinical studies, pre-clinical studies, and case reports [17]. A critical part of the general population, as well as allergic patients, use unconventional remedies, including herbal products [18,19]. Sometimes, HMPs can cause unexpected (type B) adverse events, such as allergic/hypersensitivity reactions and dose-dependent pharmacological/toxic (type A) adverse effects. Herb-drug interactions are also possible. For example, there have been reports that green tea extract can reduce the levels of fexofenadine by inhibiting the organic anion-transporting polypeptide OATP1A2-mediated intestinal absorption and that quercetin, a compound found in *Achillea millefolium*, decreases the levels and effects of fexofenadine and loratadine by inhibiting P-glycoprotein (MDR1) efflux transporter. Fortunately, such drug interactions are considered to be minor [20,21,22].

Allergic patients must be aware that ‘natural’ does not necessarily mean ‘safe’. The spectrum of hypersensitivity reactions to Asteraceae HMPs is large, both life-threatening systemic immediate type 1 ones, such as anaphylaxis, and cutaneous delayed type 4 adverse reactions, such as allergic contact dermatitis, being possible [5,20,23,24].

IgE-mediated (type 1) hypersensitivity reactions to Asteraceae HMPs may occur due to new sensitization to the herbs or, more commonly, cross-reactivity due to pre-existing IgE-mediated sensitization to weed pollen. Allergenic cross-reactivity is specified for *Chamomilla*, *Echinacea*, and other Asteraceae plants. Immediate adverse reactions to HMPs can manifest as oral allergy syndrome, urticaria, angioedema, conjunctivitis, asthma exacerbation, and even anaphylaxis [23,25,26]. According to a World Health Organization (WHO) global database, the number of reports on allergy-like reactions increased along with the use of HMPs. Commonly suspected Asteraceae plants associated with immediate reactions are *Echinacea purpurea*, along with *Matricaria recutita* and *Calendula officinalis* [27].

To effectively educate patients, it is essential to include information about Asteraceae weeds and the plants used in HMPs with cross-reactive allergenic potential, alongside conducting a proper allergy assessment.

### 3.1. HMPs from Plants Belonging to the Anthemideae Tribe in the Asteroideae Subfamily

*Matricaria chamomilla*/*Chamomilla recutita* (chamomile, also known as German chamomile, also spelled camomile) and *Chamaemelum nobile*/*Anthemis nobilis* (Roman chamomile), entomophilous herbaceous plants native to Southern Europe, are two Asteraceae representatives frequently used as HMPs. The flowers for medicinal use are obtained from cultivated or wild plants. Herbal products containing *Matricaria* flowers are usually administered as herbal infusions, more rarely as camomile enemas. The mugwort-camomile association consists of primary respiratory IgE sensitization to *Artemisia vulgaris* pollen and secondary allergic symptoms, from allergic contact conjunctivitis to anaphylaxis, in patients exposed to herbal infusion of camomile flowers. Anaphylactic reactions caused by camomile are rare, with only a few well-documented reports, but the incidence of mugwort-camomile association is considered underestimated. Some patients with allergic rhinitis to mugwort pollen may present allergic reactions to camomile, but most patients with camomile allergy are IgE-sensitized to *Artemisia* pollen. The possible cross-reactive component in this association is Art v 1 defensin, while a Bet v 1 homolog and high-molecular-weight allergens may also have a role, but not the profilin pan-allergens [6,25,28,29].

Patients who are allergic to HMPs containing camomile flos (Latin: flower) should be assessed for IgE sensitization to mugwort pollen and the allergy risks for other HMPs, honey, and bee pollen products (HBPs) consumption. Camomile may cross-react with other HMPs Asteraceae family plants, including echinacea, feverfew, and milk thistle. According to the Herbal Medicinal Products Committee (HMPC) of the EMA, *Matricaria* flower HMPs must not be used by those allergic to Asteraceae plants. Moreover, honey samples may contain pollen grains from sunflower, camomile, and yarrow [30,31,32].

*Tanacetum parthenium*/*Chrysanthemum parthenium*, known as feverfew, native to the Balkans, has yellow flowers resembling camomile, and its aerial parts are included as raw material in the European Pharmacopoeia. They may cross-react with echinacea, camomile, and milk thistle. IgE sensitization to *Tanacetum parthenium* flowers was reported in the case of mugwort pollinosis associated with anaphylaxis after drinking camomile infusion for the first time. Therefore, according to EMA’s HMPC, feverfew should not be used in patients with hypersensitivity/allergy to Asteraceae family plants [33,34,35].

*Achillea millefolium*, also known as the yarrow, is a well-known and widely distributed Asteraceae species, frequently used in HMPs as dried inflorescences for infusions. Its pollen is highly allergenic, and patients with mugwort allergic rhinoconjunctivitis may develop occupational asthma from exposure to dried yarrow flowers. According to EMA’s HMPC, yarrow flower HMPs are contraindicated in patients with known allergies to Asteraceae plants, including matricaria or marigold flowers [36,37].

*Artemisia absinthium*, or wormwood, may also cause IgE sensitization in patients with seasonal allergic rhinitis, and its leafy flowering tops may be used as HMP. Although no side effects have been reported, wormwood should not be taken by patients who are allergic to plants of the Asteraceae family [38,39].

### 3.2. HMPs from Plants Belonging to the Heliantheae Tribe in the Asteroideae Subfamily

*Echinacea purpurea* and *Echinacea angustifolia*, purple coneflowers native to North America, are two of the most well-known Asteraceae species worldwide used as HMPs due to their potential beneficial effects. *Echinacea pallida* is also utilized as HMP [40]. The flowering aerial parts of *Echinacea* spp. are available as HMPs for oral administration and can cause allergic reactions. It is clinically helpful to note that immediate pharyngeal irritation by echinacein, an isobutyl amide constituent, may cause marked local tingling and increased salivation. Relevant cases of IgE-mediated hypersensitivity reactions include urticaria, angioedema, bronchospasm, and anaphylaxis, reported even upon first known exposure. Patients allergic to *Ambrosia artemisiifolia* pollen seem more likely to present IgE sensitization to *Echinacea*, but the risk of clinically significant adverse events appears relatively low [19,26,41,42,43]. Current advice from the European Drug Safety is to caution against purple coneflower HMPs use in atopic and asthmatic patients. Due to allergic cross-reactivity risk, ragweed-allergic patients should avoid taking *Echinacea* HMPs. These should likewise not be used in patients who are hypersensitive/allergic to any plant of the Asteraceae family, according to the EMA’s HMPC [44,45].

*Helianthus annuus*, known as sunflower, has an important position as the world’s vegetable oilseed. Although historically, it was ethnomedicinally significant, presently, it is rarely used as herbal medicine in Europe. Instead, ingestion of HMPs in combination with HBPs is more common. In patients with Asteraceae anemophilous pollen allergy, especially with IgE sensitization to mugwort, HBPs were reported to induce allergic reactions due to cross-reactivity between pollen of wind-pollinated weeds and other Asteraceae insect-pollinated plants, such as sunflower and dandelion [5,46]. Moreover, in the mugwort-sunflower association, the clinical picture may include oral allergy syndrome or even anaphylaxis to ingestion of commercially peeled sunflower seeds contaminated with pollen. A similar risk in some patients sensitized to mugwort pollen is also possibly associated with the ingestion of unpeeled sunflower seeds not cleaned enough or herbal remedies with sunflower petals. Pollen grains originate from the unripe flower heads mixed during harvest with the ripe ones [6,47].

*Xanthium strumarium* (common cocklebur) and *Xanthium spinosum* (Bathurst burr or spiny cocklebur) are other Asteraceae plants used in herbal remedies in Europe, including as infusions with aerial parts. The pollen grains of cocklebur and ragweed are similar in appearance. Despite their close botanical proximity, only some published data found cross-reactivity between cocklebur and short ragweed, while others did not [48,49,50].

### 3.3. HMPs from Plants from Calenduleae and Astereae Tribes in the Asteroideae Subfamily

*Calendula officinalis*, also known as common marigold or pot marigold, is a widely cultivated plant from the Calenduleae tribe. Its flower preparations, which may be obtained by drying and comminuting, are usually used for infusions. Reported allergic adverse reactions include severe anaphylaxis after gargling with an infusion of *Calendula* and contact angioedema to pot marigold infusion in a patient with fall seasonal allergic rhinoconjunctivitis sensitized to mugwort and ragweed allergens. According to EMA’s HMPC, *Calendula* flower medicines should not be used in patients allergic to Asteraceae family plants [51,52,53].

*Solidago virgaurea*, known as European goldenrod, is a herbaceous weedy plant from the Astereae tribe, widespread across most of Europe. It is also grown as a garden flower and a familiar medicinal plant. Although it is primarily insect-pollinated, significant anemophily may occur. However, whether goldenrod is an important cause of allergic rhinitis has been debated. Cross-reactivity between goldenrod and ragweed is considered minor, but it was suggested between *Solidago*, *Matricaria,* and *Chrysanthemum* species. Goldenrod also exhibits cross-reactivity with sunflower pollen, albeit not as significant as mugwort. According to the EMA’s HMPC, HMPs with *Solidago virgaurea* are not indicated in patients with known hypersensitivity to Asteraceae family plants [25,54,55].

### 3.4. HMPs from Plants Belonging to the Cichorioideae and Carduoideae Subfamilies

*Taraxacum officinale*, known as dandelion, is a prevalent weed from the Cichorioideae subfamily and a popular folk medicine plant. Its comminuted herbal substance is used as an herbal infusion for oral use. Cross-allergenicity of pollen from dandelion with *Artemisia vulgaris* and *Chrysanthemum × morifolium* (*Dendranthema grandiflorum*) is reported. *Taraxacum* and *Chrysanthemum* spp. are frequently cosensitized with mugwort in the general population with respiratory allergies. Although these two species reveal extensive cross-allergenicity with mugwort, monosensitizations are also possible [56,57,58].

*Silybum marianum*, known as milk thistle, is a versatile Carduoideae subfamily plant adapted to different conditions worldwide. Its fruits (“seeds”) are a reliable source of silymarin, a mixture of flavonolignans, mainly silybin. Milk thistle may prompt an allergic reaction in patients with hypersensibility to Compositae plants, such as ragweed, marigolds, and chrysanthemums. The EMA’s HMPC mentions that milk thistle HMPs should not be used in patients with hypersensibility/allergy to Asteraceae family plants [58,59].

## 4. In Vivo Diagnosis Tests for IgE-Mediated Allergy

Considering the need for an accurate diagnosis of respiratory allergies with sensitization to weed pollen and the assessment of associated risks related to the use of HMPs, the allergy diagnostic workup for such patients, both in vivo and in vitro diagnosis tests, must be presented.

### 4.1. Allergy Skin Tests

Skin testing with commercial allergen extracts and non-standardized native materials represents the first-line in vivo diagnostic tool for assessing immediate type 1 hypersensitivity in clinical practice.

Epidermal skin prick testing (SPT) is an essential diagnostic tool indicated if Asteraceae IgE-mediated sensitization and allergy are suspected. The SPT provides objective confirmation of IgE sensitization, whereas the clinical relevance should always be interpreted from the perspective of detailed personal history and disease manifestations. The SPT with commercial extracts may exceptionally induce systemic reactions. Intradermal tests with Asteraceae pollen or herbal extracts are not recommended because the clinical value is not known and are less safe to perform. Skin scratch testing is also not recommended because it is difficult to interpret due to varying quantities of allergen exposure and mechanical skin irritation, with a higher risk of adverse allergic reactions [60,61,62,63]. The SPT is minimally invasive and inexpensive. Results are immediately available and reproducible when carried out by trained health professionals. The recommended method of SPT includes the appropriate use of allergen extracts, positive and negative controls, and interpretation of the tests after 15 min of application. Tests are usually applied to the volar aspect of the forearm according to European guidelines [60,61]. The positive control is histamine dihydrochloride 10 mg/mL (54.3 mmol/L), equivalent to 6.14 mg/mL histamine base, and the negative control is a saline solution, with phenol 0.5% or glycerine 50%, and no active ingredient [64].

In Europe, the SPT panel with aeroallergen extracts includes the most important Asteraceae weed pollen from short ragweed (*Ambrosia artemisiifolia*) and mugwort (*Artemisia vulgaris*), as presented in Table 1 [65,66,67,68,69].

Differences in allergen potency and concentration of major allergens among commercial SPT reagents used for the Asteraceae weed pollen allergy have been reported [70]. There is a real need for the availability of a wider range of high-quality diagnostic allergens for in vivo diagnosis of IgE-mediated allergies, covering not only predominant but also less frequent allergen sources [71,72].

We indirectly assess the HBPs’ allergic risks by SPT with *Helianthus annuus* pollen extract in patients presenting with Asteraceae anemophilous pollen allergy who were not significantly previously exposed to sunflower pollen in surroundings of farmlands with cultivated sunflowers or in agricultural occupational settings [5,46].

Different Asteraceae inflorescences utilized as HMPs may be used for SPT as pure floral liquid extracts, hot water flower infusions after cooling, or suspension of comminuted, dried inflorescences or powdered capitulum in sterile saline solution at room temperature. Native floral products used for SPT are obtained from infusion bags purchased in a local retail shop or brought by the patient from the herbal box used before the reaction.

A camomile extract 1:10 *w*/*v* used for SPT may be obtained from 2 g of dried comminuted or pulvis of *Matricaria chamomilla* flowers, defatted with acetone, and extracted in 20 mL of 0.01 mol/L of phosphate and 0.15 mol/L of NaCI (phosphate buffered saline). The mixture is then stirred for 24 h at 4 °C, centrifuged for 20 min, and passed through a paper filter with 0.22 μm pores. The SPTs begin with a 1:100,000 *w*/*v* concentration, then 1:10,000 *w*/*v*, 1:1000 *w*/*v* and 1:100 *w*/*v* camomile extracts. It is important to note that control nonatopic subjects consistently do not react to camomile infusion extract 1:10 *w*/*v* or 10% *w*/*v* solution [73,74].

Hot water camomile infusion for SPT may be used as a 3% *w*/*v* solution, equivalent to 30 mg/mL. Reports were published either with SPT with brewed commercial camomile infusion obtained by soaking 3 g flowers with 100 mL boiled water for 10 min cooled before testing [29] or with SPT with hot water camomile infusion prepared by pouring 3 g pure natural, ready-filled, dried camomile flowers into 250 mL of water at a rolling boil, allowed to infuse for 5 min and then to cool down to room temperature slowly [28,75]. The SPT may also be performed with a commercial oily extract of manzana camomile flowers 98.91% [76] or camomile commercial infusion 10 mg/mL prepared by mixing 1 g of dried flowers in 100 mL of sterile saline for 30 min [77]. *Calendula officinalis* flores 30 mg/mL pulvis suspension obtained by mixing dried pot marigold flowers in sterile saline may also be used for SPT [53]. SPTs with aqueous extracts from dried flowers of yarrow *Achillea millefolium*, aqueous *Echinacea* solutions, extracts prepared from *Echinacea* aerial parts in increasing concentrations, and glycerinated *Echinacea* extracts obtained from an HMP manufacturer were published [26,36,41]. Moreover, SPT with components of non-Asteraceae herbal medicines may be additionally needed if combined or complex herbal products are suspected [78].

An alternative in vivo diagnostic approach is the prick-prick test (PPT) with native allergens, which is practical and less resource-consuming [79]. The PPT with soaked flowers in normal saline may be performed. Central disk tubular florets and peripheral ray or ligulate florets may be used separately. A PPT was reported with non-heated central tubular florets, where most pollen adhered, and ligulate florets of feverfew inflorescence [35].

### 4.2. Allergen Provocation Tests

Allergen challenges are in vivo diagnostic tools offering reproducible data in patients with pollen allergy using standardized allergen extracts applied to the nasal or bronchial mucosa to induce symptoms according to European guidelines for the purpose of demonstrating the clinical relevance of IgE-mediated sensitization identified by SPT and in vitro immunoassays. The most frequently applied airway allergen provocation is the nasal allergen challenge (NAC). There is evidence of a relationship between positive nasal challenges and ocular reactions. The conjunctival allergen provocation test, which instills the allergen solution on the ocular surface, is less frequently used. Moreover, the bronchial allergen challenge (BAC) is usually considered a research tool only, and its reproducibility was not evaluated for weed pollen allergens. All these provocation tests are instrumental in confirming the allergy diagnosis if detailed clinical history, SPT, and specific IgE findings are inconclusive, considering the potential for systemic adverse reactions, especially with inhalation challenges [79,80,81,82]. Examples of Asteraceae weed pollen allergen extracts used as individual diagnostic allergens for NAC and BAC, commercially available in some European countries, are presented in Table 2 [25,36,67,83,84]. All in vivo allergy provocation tests require highly qualified staff and safety measurements [79,81,82].

The NAC is a valuable diagnostic and research tool for evaluating the response of the nasal mucosa to aeroallergens with high sensitivity and specificity. It is the gold standard in the diagnosis of local allergic rhinitis. It can be used to diagnose seasonal allergic rhinitis, especially to detect clinically relevant allergens in polysensitized patients or those with discrepancies between clinical history, SPT, and specific IgE results. NAC also offers valuable information in evaluating asthmatic patients since 85% of patients reveal identical responses at the nasal and bronchial mucosa upon allergen provocation. The advantage of NAC is that it is a much safer procedure than BAC [85,86].

Allergen exposure chambers (AECs) are modern specialized medical installations designed for patients’ standardized and reproducible allergen challenges. They mimic real-world natural exposure to aeroallergens while allowing for precise control over the allergen concentration and other environmental variables. Until recently, one study has been published using a ragweed AEC model to assess immunotherapy preparation [87,88,89].

Oral provocation tests with Asteraceae HMPs liquid preparation are performed in clinical practice only after a carefully assessed risk/benefit ratio.

A labial provocation test was carried out by applying one drop of undiluted camomile infusion on the lip, obtained from 3 g flowers with 100 mL boiled water for 10 min, and cooled before testing. An open oral challenge may follow starting at 1:100,000–1,000,000 dilution of herbal infusion prepared as usual and increased 10-fold to full strength at 15–20 min intervals, using a 5 mL volume. If there is no reaction after full strength, ad-lib drinking under observation may be suggested [9,63].

## 5. In Vitro Diagnosis Tests for IgE-Mediated Allergy

Immunoassays for specific IgE are the most widely used in vitro tests for IgE-mediated allergy to Asteraceae weed pollen and HMPs. BAT is an emerging additional method.

### 5.1. Serum Specific IgE (ssIgE)

The measurement of ssIgE antibodies against natural allergen extracts and molecular components, either well-defined highly purified natural or recombinant molecules, is an essential complementary in vitro tool to diagnose IgE-mediated sensitization to Asteraceae pollen, especially in subjects who cannot undergo SPT, such as those with dermographism or who are taking H1 antihistamines or other medications that interfere with the proper interpretation of results [61]. Either ssIgE or SPT are acceptable for diagnosing respiratory allergies and guiding allergen immunotherapy [90].

Immunoassays used to detect ssIgE to allergen extracts and components are either singleplex, multiparameter, or multiplex assays, depending on the number of allergen extracts and molecular components used [91].

The radioallergosorbent test (RAST) was the first generation isotopic singleplex immunoassay that used allergens covalently coupled to an allergosorbent filter paper solid phase, radioiodinated polyclonal anti-IgE, and bound radioactivity quantification by gamma counter. The ssIgE were detected by this method in a case of anaphylaxis to *Echinacea* sp. Such ssIgE in asymptomatic atopic subjects never exposed to oral HMPs or ornamental *Echinacea* suggests cross-reactivity between structurally related allergens in *Echinacea* and other Asteraceae pollen grains [91,92].

Current commercial singleplex, nonisotopic, more sensitive in vitro methods for ssIgE to many natural extracts and molecular components use either solid-phase coupled allergens (i.e., fluorescence enzyme immunoassay) or liquid-phase allergens (i.e., chemiluminescence immunoassay). Asteraceae pollen whole allergens as native extracts (Table 3) are obtained from the pollen grains of many entomophilous weeds and primarily anemophilous plants used as HMPs [91,93,94,95]. When compared with multiplex technology, the advantages of singleplex assays for allergen ssIgE testing with allergenic molecules/components include increased sensitivity at low ssIgE levels [91].

The fluorescence enzyme immunoassay (FEIA) with capsulated cellulose polymer solid-phase coupled allergens (ImmunoCAP^®^, Thermo Fisher Scientific Inc., Phadia AB, Uppsala, Sweden) is a currently used singleplex assay to measure ssIgE to Asteraceae pollen allergens. Allergens are covalently coupled in FEIA to a hydrophilic carrier polymer consisting of a cyanogen bromide-activated cellulose derivative with a large surface for protein binding. This method uses β-galactosidase-labeled anti-IgE monoclonal antibodies and 4-methylumbelliferyl-β-galactoside as fluorogenic substrate, fluorescence measurement being performed with a fluorocounter [91,96].

The enzyme-enhanced chemiluminescence immunoassay (CLIA) with liquid-phase allergens is another advanced singleplex detection method for ssIgE that exploits liquid-phase kinetics in a bead format (3gAllergy™ Immulite^®^ 2000 and Immulite^®^ 2000 XPi immunoassay; Siemens Healthcare Diagnostics Inc., Erlangen, Germany). Asteraceae allergens covalently bound to soluble biotinylated polylysine polymer in a fluid phase bind to streptavidin-coated polystyrene bead in the reaction tube (through a streptavidin-biotin interaction). This method uses alkaline phosphatase enzyme-labeled anti-IgE monoclonal antibodies and adamantyl 1,2-dioxetane aryl phosphate as chemiluminescent substrate, chemiluminescence being measured using a luminometer [91].

In the past, patients with ssIgE concentrations of 0.35 kU_A_/L or higher were considered to have a positive result. However, more recently, ssIgE tests have been reported as analytical results, not based on positive or negative thresholds. Also, it was suggested that the lowest detectable limit for autoanalyzer-based IgE immunoassays should be 0.1 kU_A_/L. Nevertheless, ssIgE levels between 0.1 and 0.35 kU_A_/L should be carefully considered along with the clinical manifestations. It is essential to distinguish between IgE sensitization and allergy at all concentrations. Elevated ssIgE values alone, without characteristic symptoms, are insufficient for a definitive allergy diagnosis [97].

A multiparameter line blot immunoassay with allergens coating membrane strips in thin parallel lines as line blots (Euroline™; Euroimmun AG, Lübeck, Germany) may be used to measure simultaneously ssIgE against several allergen extracts, including common ragweed (w1) and mugwort (w6) pollen. This in vitro diagnosis method based on immunoblot technology uses alkaline phosphatase enzyme-labeled anti-IgE monoclonal antibodies and nitroblue tetrazolium chloride/5-bromo-4-chloro-3-indolylphosphate for colorimetric detection, with subsequent image acquisition and evaluation. If anti-CCD IgE antibodies are detected in serum reflected in a positive CCD marker band, the serum should be re-incubated in the assay with anti-CCD absorbent [91,98].

Multiplex ssIgE immunoassays enable the detection of the profile of IgE sensitizations against a wide array of allergens (more than one hundred from various sources). Asteraceae pollen allergens are present as native extracts and molecular components (Table 4) [93,94]. The microarray immunoassay on a preactivated amine-reactive polymer-coated glass slide as a solid phase for allergen components uses fluorophore-labeled anti-human IgE monoclonal antibodies (Immuno CAP^®^ ISAC™, Thermo Fisher Scientific Inc., Phadia AB). The ELISA-based macroarray immunoassay uses nano-bead technology as a molecular allergy explorer (ALEX^®^, MacroArray Diagnostics, Vienna, Austria) with allergen extracts and molecular components spotted in a cartridge chip onto a nitrocellulose membrane, using anti-human IgE labeled with alkaline phosphatase. Its protocol integrates a potent CCD inhibitor during serum incubation, thus increasing the specificity of the test results [91,96].

Because pollen allergy is heterogeneous regarding clinical manifestations and sensitization profiles, including poly-sensitization, molecular customized in vitro IgE immunoassays can detect relevant sensitizations [99].

Asteraceae weed pollen allergen components used in singleplex immunoassays are ragweed pectate lyase Amb a 1, mugwort defensin-like protein Art v 1, and non-specific lipid transfer protein Art v 3 (Table 3).

A useful diagnostic marker for genuine IgE sensitization to *Ambrosia artemisiifolia* pollen is ssIgE to major allergen Amb a 1. This pectate lyase is responsible for more than 90% of the IgE responses in short ragweed pollen-allergic patients. Amb a 1.01 is its most allergenic isoform. Although it shows cross-reactivity with mugwort Art v 6 and sunflower Hel a 6, in areas of concomitant exposure to ragweed and mugwort with overlapping pollination seasons, Amb a 1 assists with the identification of the primary sensitizer [100,101]. The cysteine protease Amb a 11 is also considered a major ragweed pollen allergen with an IgE sensitization rate of up to almost 70%; however, sensitization studies in larger cohorts remain to be conducted [102,103].

Several less frequently recognized allergens are considered relevant, such as defensin Amb a 4, non-specific lipid transfer protein type 1 Amb a 6, and profilin Amb a 8, due to their ability to cross-react with other homologous allergens, such as Art v 1 (with Amb a 4), Art v 3 (with Amb a 6), and profilin pan-allergens from Timothy grass Phl p 12 and birch pollen Bet v 2 (with Amb a 8) [1,6,104].

Amb a 4 is a minor allergen from *Ambrosia* pollen present in multiplex macroarray immunoassay but with limited quantity in ragweed extracts. The IgE binding frequency to Amb a 4 is about 20% in ragweed-allergic patients and even higher in mugwort-cosensitized populations. Among weed pollen-allergic patients, positive IgE reactivity to Amb a 4 may be up to 40%, and it is typically associated with Art v 1 sensitization. DPLP Amb a 4 is an Art v 1 homolog protein with a defensin-like domain, but its proline-rich region differs from Art v 1, affecting the IgE binding. Thus, Amb a 4 has different degrees of cross-reactivity with the major mugwort pollen allergen Art v 1. Amb a 4 is also responsible for primary sensitization in some patients with high ragweed exposure [1,103,105].

Other *Ambrosia* pollen allergen components not yet included in commercial assays, such as plastocyanins Amb 3 and Amb a 7, polcalcins or calcium-binding proteins Amb a 9 and Amb a 10, and enolase Amb a 12, may also contribute to the molecular IgE sensitization profiles of patients with respiratory allergy to ragweed pollen [106].

A prominent diagnostic marker for genuine IgE sensitization to *Artemisia vulgaris* pollen is ssIgE to common mugwort major allergen Art v 1. This defensin-polyproline-linked protein, also named defensin-like protein linked to polyproline-rich region (DPLP), is recognized by more than 70% of mugwort-sensitized patient IgE antibodies. Native Art v 1 (nArt v 1) was revealed to have higher reactivity than the recombinant Art v 1 (rArt v 1) due to the involvement of carbohydrate-specific antibodies in epitope recognition. The wide-ranging carbohydrate structures (representing 30–40% of the nArt v 1) with C-terminal hydroxyprolines revealing different degrees of *O*-glycosylation are important in IgE recognition. Although rArt v 1 produced in *E. coli* lacks the posttranslational modifications present in nArt v 1, both share common epitopes recognized by human IgE antibodies. While nArt v 1 is a complex mixture of different isoallergens and glycosylation variants, rArt v 1.0101 is considered a relevant mugwort pollen isoallergen [1,103,105,107]. There is extensive cross-reactivity among the major allergens of different species of mugwort (more than 94% protein sequence identity). Pollen grains from Asteraceae plants botanically related, such as sunflower and wormwood, possess Art v 1 cross-reactive structures. Art v 1-like molecules are responsible for the cross-reactivity of mugwort with sunflower and camomile [107,108].

Art v 6 also plays a crucial role in mugwort pollen allergy, and the cross-reactivity between Art v 6 and Amb a 1 is frequent, bidirectional, and relevant. Double sensitization to ragweed and mugwort is common in many areas. Cross-reactivity is responsible for double sensitization in most cases. In some regions, if individuals sensitized to profilins and polcalcins are excluded, both IgE sensitization and allergy to mugwort pollen in the absence of ragweed sensitization are rare. A practical approach was suggested for patients with IgE sensitization to both mugwort and ragweed extracts reactive to Art v 1 to be prescribed mugwort immunotherapy, whereas those not reactive to Art v 1 to be prescribed ragweed immunotherapy because *Ambrosia* is probably the primary sensitizer [109].

The nsLTP Art v 3 is a marker allergen for complex sensitization profiles to mugwort pollen. Patients with allergic rhinitis to *Artemisia vulgaris*, living in areas with high mugwort pollen exposure, exhibit more complex sensitization profiles, including more frequent Art v 3 sensitization, which is associated with an increased prevalence of allergic asthma [1,108]. The relevant isoallergen rArt v 3.0201 possesses similar structural and immunological properties as nArt v 3, enabling batch-to-batch reproducibility for a high-quality diagnosis [110]. Moreover, this PR-14 protein is also a marker allergen for nsLTP pan-allergen sensitization. Art v 3 cross-reactivities with homologous peach Pru p 3 from hazelnut Cor a 8 and other nsLTPs are involved in some pollen-food syndromes, including mugwort-peach and Asteraceae-hazelnut associations [6,16,53,91].

Other cross-reactive allergen components, such as PR-1 protein Art v 2, profilin Art v 4, polcalcin Art v 5, may also contribute to the molecular IgE sensitization profiles in mugwort respiratory allergy [106,111]. Some authors suggested that using Art v 1 and Art v 4 could identify 91% of mugwort pollen-sensitized patients [112].

Because polysensitizations to different pollen sources are found in about 20% of pollen-allergic subjects, molecular diagnosis is needed to distinguish broad cross-reactivity due to pan-allergen profilins such as Bet v 2 or Phl p 12 and/or polcalcins such as Bet v 4 or Phl p 7, from primary IgE sensitizations to major marker allergens [113].

Moreover, group 1 and 4 allergen molecules of grass pollen are significant inducers of carbohydrate cross-reactive determinants (CCDs), moieties of glycoproteins that induce highly cross-reactive IgE antibodies. CCDs do not usually induce mast cell degranulation in vivo and, therefore, do not elicit positive reactions to SPT with allergen extracts or symptoms upon allergen exposure. However, the presence of CCDs may cause false-positive results in IgE immunoassays with CCD-containing natural allergen extracts from pollen, plant foods, and hymenopteran venoms. CCD inhibition improves the accuracy of in vitro detection of pollen allergen-specific IgE [1,114,115].

### 5.2. Specific IgE in Nasal Secretions (nssIgE)

Although some published research data suggested that nssIgE against aeroallergens seems to be a non-specific phenomenon since they can also be detected in nonallergic rhinitis and healthy subjects [116], several novel studies suggested using nssIgE as an additional local allergic rhinitis (LAR) diagnostic criterion [117]. A characteristic feature of LAR is the presence of nssIgE in 20–40% of subjects [118]. If the nasal cytological test reveals a prominent accumulation of eosinophils in nasal mucosa without detectable levels of ssIgE, local levels of nssIgE may be used to support the diagnosis of LAR as an alternative to NAC [97].

The nssIgE against mugwort pollen may be determined using FEIA with capsulated cellulose polymer solid-phase coupled allergens (ImmunoCAP^®^, Thermo Fisher Scientific Inc., Phadia AB, Uppsala, Sweden). Nasal secretions may be obtained using a sinus sponges pack in each nostril or bilateral nasal lavage performed with physiologic saline solution at room temperature. The detection limit is 0.35 kU_A_/L. Measurements are performed at baseline and 15 min and 1 h after the NPTs [119,120].

### 5.3. Basophil Activation Test (BAT)

The Flow CAST (Cellular Allergy Stimulation Test) is a standardized BAT (Bühlmann Laboratories AG, Schönenbuch, Switzerland) may be used in clinical research and diagnostic labs for the quantitative assessment of in vitro basophil activation by common ragweed and mugwort allergens, by flow cytometry performed using the FACSCalibur^®^ system (Becton-Dickinson Immunocytometry System, Heidelberg, Germany). The chemokine receptor CCR3 is used as an identification marker for basophils, while CD63 is used as a basophil activation marker, labeled with anti-CCR3-phycoerythrin and anti-CD63-fluorescein-isothiocyanate monoclonal antibodies, respectively. BAT is considered a functional ex vivo assay differentiating sensitization and relevant allergy. Basophil allergen threshold sensitivity (CD-sens) has been shown to correlate with NAC and BAC threshold sensitivity [96,121,122]. The utility of BAT in weed pollen allergy was mentioned in patients with mugwort immunotherapy [123] and mugwort pollen-related peach allergy [124]. The usefulness of BAT for bupleurum-containing Japanese herbal medicine-induced lung injury was recently published. To our knowledge, there are no other reports of BAT with HMPs in Europe [125].

## 6. Conclusions

To optimize the management of patients with respiratory allergies to Asteraceae weed pollen, general physicians, ENT specialists, and pulmonologists need to be updated on the diagnostic methods allergists use in clinical practice and their usefulness and limitations. A modern molecular allergology approach relying on utilizing allergen molecules as in vitro tools that complements the traditional approach based on SPT and in vitro allergen extract testing [126] may assess IgE sensitization to specific weed pollen allergens and cross-reactive allergen components in HMPs, including Asteraceae plant defensins [6,25,28,29] with good thermal and pH stability [127]. Allergists also need to become more knowledgeable about herbal therapies so that they can educate patients about the potential risks of HMPs. It is relevant to know that HMPs containing Asteraceae herbs (flowering aerial parts) preparations are usually available for oral administration in solid or liquid forms. Herbal infusions are the most frequently used liquid products. They are either loose or obtained from single-use infusion bags, considering that Asteraceae pollen grains are smaller than the pore size of woven nylon and non-woven (cellulosic, polylactic acid, nylon hybrid) infusion papers. Enemas with herbal liquid preparation are even more risky due to application to a large area of colonic mucosa [76,128,129]. Allergists must inform patients allergic to weed anemophilous pollen, such as from mugwort or ragweed, about the allergic risks of flowers/aerial parts of entomophilous Compositae plants used as HMPs consumed without or with HBPs, which may contribute to additional risks.

## Figures and Tables

**Table 1:** Examples of Asteraceae weed pollen allergen extracts used as individual diagnostic allergens for SPT in the EU.

Skin Prick Test Allergen Solutions *	Potency/Concentration **	Marketing Authorization ***
Mugwort Soluprick^®^ SQ 312	10 HEP/mL	ALK-Abelló
Ragweed (*Ambrosia*) Soluprick^®^ N 302	1:100 *w*/*v* (10 mg/mL)	ALK-Abelló
Dandelion prick test solution 143	50,000 BU/mL	Allergopharma
Mugwort prick test solution 106	50,000 SBU/mL	Allergopharma
Ragweed prick test solution 154	50,000 BU/mL	Allergopharma
Dandelion prick test solution	10,000 DU/mL	Bencard Allergy
Mugwort prick test solution	10,000 DU/mL ODC	Bencard Allergy
Ragweed prick test solution	10,000 DU/mL	Bencard Allergy
Mugwort prick test solution	30 HEP/mL	Leti Pharma
Mugwort pollen prick test solution	10,000 AU/mL	HAL Allergy
Ragweed (*Ambrosia*) pollen prick test solution	10,000 AU/mL	HAL Allergy
Mugwort (*Artemisia vulgaris*) Alyostal^®^	100 IR/mL	Stallergenes
Ragweed (*Ambrosia elatior*) Alyostal^®^	100 IR/mL	Stallergenes

Note: * weed pollen allergen products authorized in Germany included in the database of the Paul-Ehrlich-Institut, with the exception of Alyostal prick solutions with pollen allergen extracts from common mugwort (*Artemisia vulgaris*) and short ragweed (*Ambrosia artemisiifolia* var. *elatior*) commercially available in other EU countries; other prick test solutions used in some EU countries include mugwort and ragweed prick tests Bial Aristegui/Roxall, and skin prick tests Lofarma with individual Asteraceae pollen extracts from mugwort, short ragweed, giant ragweed (*Ambrosia trifida*), wormwood (*Artemisia absinthium*), sunflower (*Helianthus annuus*). In the USA, there are commercially available additional Stallergenes Asteraceae pollen extracts for SPT besides those from short ragweed and mugwort, namely from giant ragweed (*Ambrosia trifida*), sagebrush (*Artemisia tridentata*), prairie sage (*Artemisia ludoviciana*), lanceleaf ragweed (*Ambrosia bidentata*), western ragweed (*Ambrosia psilostachya*), burrobrush (Ambrosia salsola), rabbit bush (*Ambrosia deltoidea*), false ragweed (*Ambrosia acanthicarpa*), dandelion (*Taraxacum officinale*) and sunflower. ** Potency/concentration of allergen preparations expressed in different proprietary units used by each allergen manufacturer: HEP = Histamine Equivalent in Prick testing; *w*/*v* = weight/volume; SBU = Standardized Biological Units, BU = Biological Units; DU = Diagnostic Units, ODC = Optimal Diagnostic Concentration; AU = Allergy Units; IR = Index of Reactivity; *** Marketing Authorization Holder name.

**Table 2:** Examples of Asteraceae weed pollen allergen extracts used as individual diagnostic allergens for nasal and bronchial allergen provocation in some European countries.

Provocation Allergen Solution *	Potency **	Authorization ***
Dandelion provocation test solution for nasal spray	5000 BU/mL	Allergopharma
Mugwort provocation test solution for nasal spray	5000 SBU/mL	Allergopharma
Ragweed provocation test solution for nasal spray	5000 BU/mL	Allergopharma
Ragweed provocation test solution for nasal spray (Allergy Provo Spray)	10,000 AU/mL	HAL Allergy
Mugwort provocation test solution for nasal spray (Allergy Provo Spray)	10,000 AU/mL	HAL Allergy
Mugwort provocation test solution for nebulizer (Allergy Provo Test ****)	10,000 AU/mL	HAL Allergy
Mugwort provocation test (powder for solution) for nasal spray/nebulizer	30 HEP/mL	Leti Pharma

Note: * weed pollen allergen products authorized in Germany included in the database of the Paul-Ehrlich-Institut. ** Potency/concentration of allergen preparations expressed in different proprietary units: BU = Biological Units, SBU = Standardized Biological Units; AU = Allergy Units; HEP = Histamine Equivalent units. *** Marketing Authorization Holder name. **** May also be used for conjunctival provocation; BAC was performed in individual cases with aqueous extracts of camomile *Matricaria chamomilla* and yarrow *Achillea millefolium* (1.25 mg/mL).

**Table 3:** Examples of Asteraceae pollen individual diagnostic allergens in singleplex IgE immunoassays.

Asteraceae Allergen	Latin Name, Protein Group	Code	Singleplex Method	
Asteraceae pollen whole allergens (native extracts)				
Common ragweed	*Ambrosia artemisiifolia* (*A. elatior*)	w1	ImmunoCAP^®^ FEIA	Immulite^®^ CLIA
Western ragweed	*Ambrosia psilostachya*	w2	ImmunoCAP^®^ FEIA	Immulite^®^ CLIA
Giant ragweed	*Ambrosia trifida*	w3	ImmunoCAP^®^ FEIA	Immulite^®^ CLIA
False ragweed	*Ambrosia acanthicarpa*	w4	ImmunoCAP^®^ FEIA	Immulite^®^ CLIA
Wormwood	*Artemisia absinthium*	w5	ImmunoCAP^®^ FEIA	Immulite^®^ CLIA
Mugwort	*Artemisia vulgaris*	w6	ImmunoCAP^®^ FEIA	Immulite^®^ CLIA
Marguerite/Ox-eye daisy	*Chrysanthemum leucanthemum*	w7	ImmunoCAP^®^ FEIA	Immulite^®^ CLIA
Dandelion	*Taraxacum vulgare*	w8	ImmunoCAP^®^ FEIA	Immulite^®^ CLIA
Cocklebur	*Xanthium strumarium*/*commune*	w13	ImmunoCAP^®^ FEIA	Immulite^®^ CLIA
Rough marshelder	*Iva annua*/*ciliata*	w16	ImmunoCAP^®^ FEIA	Immulite^®^ CLIA
Rabbit bush	*Ambrosia deltoidea*	w36		Immulite^®^ CLIA
Common sagebrush	*Artemisia tridentata*	w43		Immulite^®^ CLIA
Groundsel bush	*Baccharis halimifolia*	w67		Immulite^®^ CLIA
Sunflower	*Helianthus annuus*	w204	ImmunoCAP^®^ FEIA	
Camomile/chamomille	*Matricaria chamomilla*	w206	ImmunoCAP^®^ FEIA	
Asteraceae weed pollen allergen components				
nAmb a 1 ragweed	pectate lyase *Ambrosia artemisiifolia*	w230	ImmunoCAP^®^ FEIA	
nArt v 1 mugwort	DPLP *Artemisia vulgaris*	w231	ImmunoCAP^®^ FEIA	Immulite^®^ CLIA
nArt v 3 LTP mugwort	nsLTP type 1 *Artemisia vulgaris*	w233	ImmunoCAP^®^ FEIA	

Note: FEIA = fluorescence enzyme immunoassay, CLIA = chemiluminescence immunoassay; DPLP = defensin-polyproline-linked protein (a defensin-like protein linked to polyproline-rich region), nsLTP = non-specific lipid transfer protein; n = natural purified, r = recombinant.

**Table 4:** Examples of Asteraceae pollen allergen extracts and components used in multiplex IgE immunoassays.

Asteraceae Allergen	Latin Name, Protein Group	Code	Singleplex Method
Asteraceae pollen whole allergens (native extracts)			
Common ragweed	*Ambrosia artemisiifolia* (*A. elatior*)	w1	ALEX2^®^
Mugwort	*Artemisia vulgaris*	w6	ALEX2^®^
Asteraceae weed pollen allergen components			
nAmb a 1 ragweed	pectate lyase *Ambrosia artemisiifolia*	w230	ImmunoCAP^®^ ISAC™ E112i
rAmb a 1 ragweed	pectate lyase *Ambrosia artemisiifolia*	w230	ALEX2^®^
rAmb a 4 ragweed	plant defensin/DPLP *Ambrosia artemisiifolia*	w300	ALEX2^®^
nArt v 1 mugwort	plant defensin/DPLP *Artemisia vulgaris*	w231	ImmunoCAP^®^ ISAC™ E112i
rArt v 1.0101 mugwort	plant defensin/DPLP *Artemisia vulgaris*	w231	ALEX2^®^
nArt v 3 LTP mugwort	non-specific LTP type 1 *Artemisia vulgaris*	w233	ImmunoCAP^®^ ISAC™ E112i
rArt v 3.0201 LTP mugwort	non-specific LTP type 1 *Artemisia vulgaris*	w233	ALEX2^®^

Note: ALEX2^®^ is the improved successor product of ALEX^®^ = Allergy Xplorer ELISA-based macroarray immunoassay, ImmunoCAP^®^ ISAC™ = Immuno Solid-Phase Allergen Chip microarray immunoassay; DPLP = defensin-polyproline-linked protein (defensin-like protein linked to polyproline-rich region), LTP = lipid transfer protein; n = natural purified, r = recombinant; Both multiplex immunoassays include pan-allergens from Timothy grass pollen: polcalcin marker rPhl p 7 and profilin marker rPhl p 12.
